# A new triclinic polymorph of 6,6′-{(1*E*,1*E*′)-[(1,2-di­phenyl­ethane-1,2-di­yl)bis­(aza­neylyl­idene)]bis­(methane­ylyl­idene)}bis­(2-chloro­phenol)

**DOI:** 10.1107/S2056989025008850

**Published:** 2025-10-17

**Authors:** Marika Iwatani, Daisuke Nakane, Takashiro Akitsu

**Affiliations:** a Department of Chemistry, Faculty of Science, Tokyo University of Science, 1-3 Kagurazaka, Shinjuku-ku, Tokyo 162-8601, Japan; University of Hyogo, Japan

**Keywords:** crystal structure, polymorph, salen-type compound, intra­molecular hydrogen bonding

## Abstract

The redetermined structure of a new polymorph of 6,6′-{(1*E*,1*E*′)-[(1,2-di­phenyl­ethane-1,2-di­yl)bis­(aza­neylyl­idene)]bis­(methane­ylyl­idene)}bis­(2-chloro­phenol), C_28_H_22_Cl_2_N_2_O_2_ was reported. The title compound was synthesized under microwave irradiation at 90 K, within 10 min. The asymmetric unit comprises a potentially tetra­dentate Schiff base ligand that crystallizes in the triclinic system with space group *P*1. The structure displays intra­molecular O—H⋯N hydrogen bonding, forming an *S*(6) ring. Comparisons are made with the monoclinic polymorph [Shen *et al.* (2017). *RSC Adv.***7**, 40640–40649].

## Abstract 2

The polymorphism of the title salen-type compound synthesized from *racemic*-(+/-)-1,2-di­phenyl­ethyl­enedi­amine and 3-chloro­salicyl­aldehyde is reported

## 1. Chemical context

Schiff base complexes have played a central role in the development of coordination chemistry, as evidenced by their vast number, ease and flexibility of synthetic procedures, diverse properties, and applications as bioactive compounds such as anti­tumor, anti­bacterial, anti­fungal, and various other biological applications. Numerous reports have shown that salen-type metal complexes exhibit high activity against various diseases, including cancer (Nworie *et al.*, 2016 ▸). Polymorphism is known to exist in crystals of Schiff base complex ligands and, as reported by Suda *et al.* (2021 ▸), in particular the angle of the phenyl group bonded to the nitro­gen atom was different from that previously reported. Salen-type compounds are synthesized from di­amines and salicyl­aldehyde and contain two azomethine groups that are used as organic ligands in complex formation (Akitsu *et al.*, 2011 ▸). In our laboratory, we have been studying salen-type metal complexes, which have the potential to be useful in a variety of applications, including as a new concept dye for dye-sensitized solar cells (DSSC), flame retardant in heat-stabilized PVC sheets and artificial metalloenzymes that mimic the catalytic efficiency of natural metalloenzymes (Yamane *et al.*, 2017 ▸; Soni *et al.*, 2020 ▸; Kashiwagi *et al.*, 2019 ▸). Salen-type metal complexes are known to exhibit ligand disorder (Akitsu *et al.*, 2005*b* ▸). However, there are also examples of non-disordered salen-type metal complexes (Akitsu *et al.*, 2005*a* ▸). In this report, we describe a new polymorphic crystal structure of the title compound.

## 2. Structural commentary

**Figure 1:**
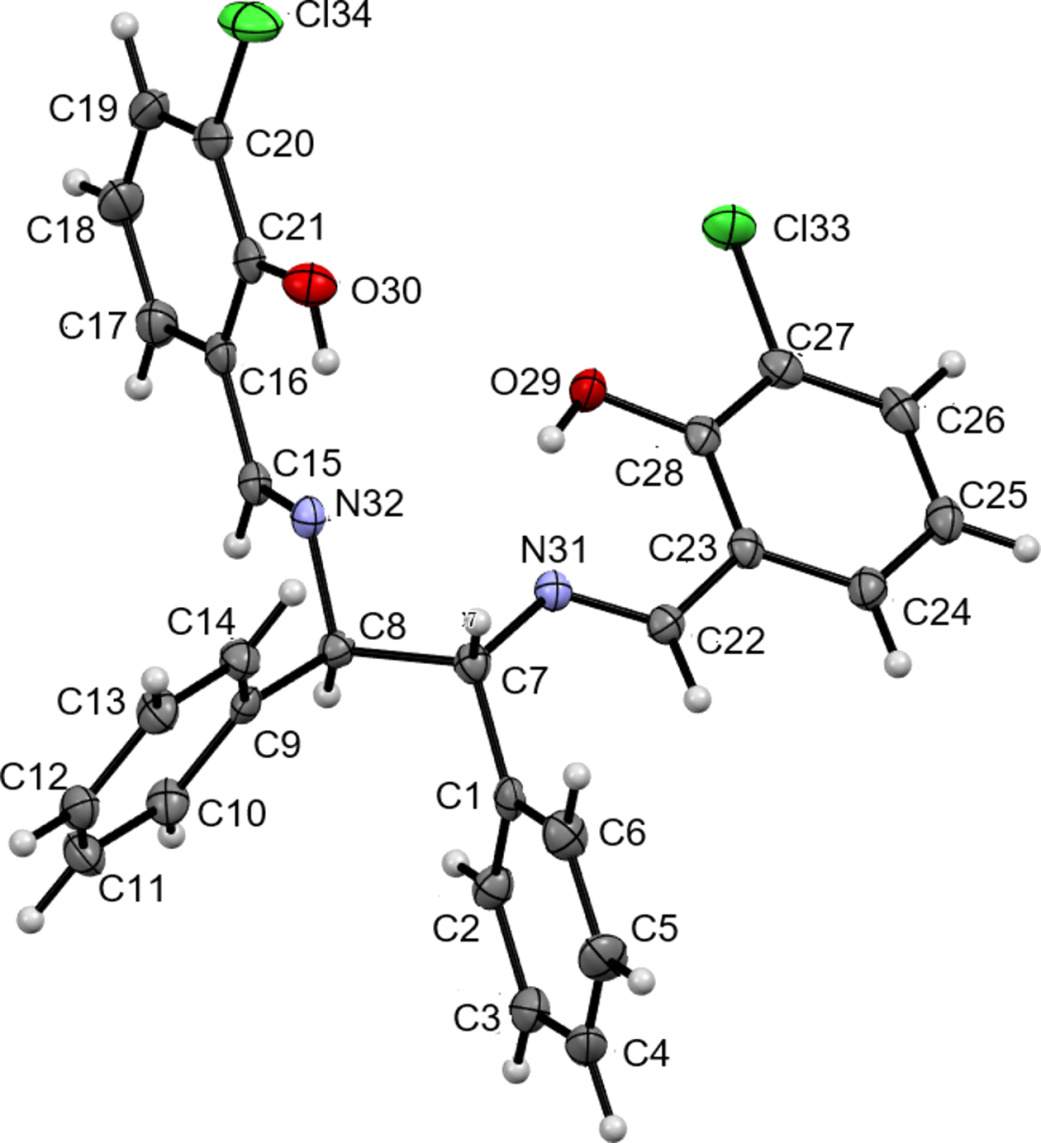
The title compound with ellipsoids drawn at the 50% probability level.

The title compound (Fig. 1 ▸) crystallizes in the triclinic system with *P*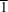 space group. The crystals obtained were racemic, with one mol­ecule being an *RR* isomer (optically active). The previously reported crystal was monoclinic, had a space group of *I*2/a, and was colorless and transparent (Shen *et al.*, 2017 ▸). Intra­molecular hydrogen bonds with an *S*(6) (Bernstein *et al.*, 1995 ▸) ring formation are observed, with O⋯N distances of 2.564 (3) and 2.597 (3) Å (Table 1 ▸).
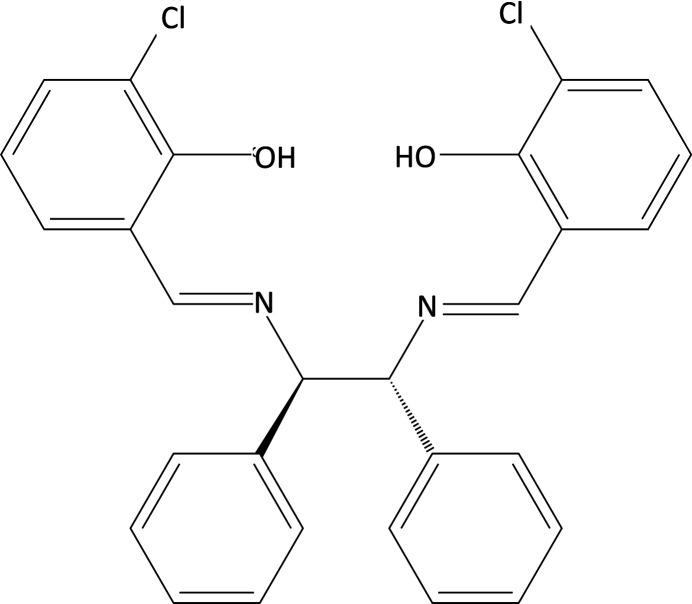


Comparing the bond distances, bond angles, and torsion angles with monoclinic polymorph the bond distances and bond angles are almost the same, but there are significant differences in the torsion angles. The torsion angles C6—C7—N31—C22 and C9—C8—N32—C15 are −22.8 (3) and −105.8 (2)°, respectively, in the title compound compared 108.3 (2)° in the monoclinic polymorph. In the title compound, N32—C8—C7—N31 is −50.7 (2)° [43.3 (2) and −42.6 (2)° in the monoclinic polymorph] while N32—C8—C9—C14 and N31—C7—C6—C1 are −60.7 (3) and −72.1 (3)°, respectively [43.2 (2) and −85.8 (2)° in the monoclinic polymorph].

## 3. Supra­molecular features

**Figure 2:**
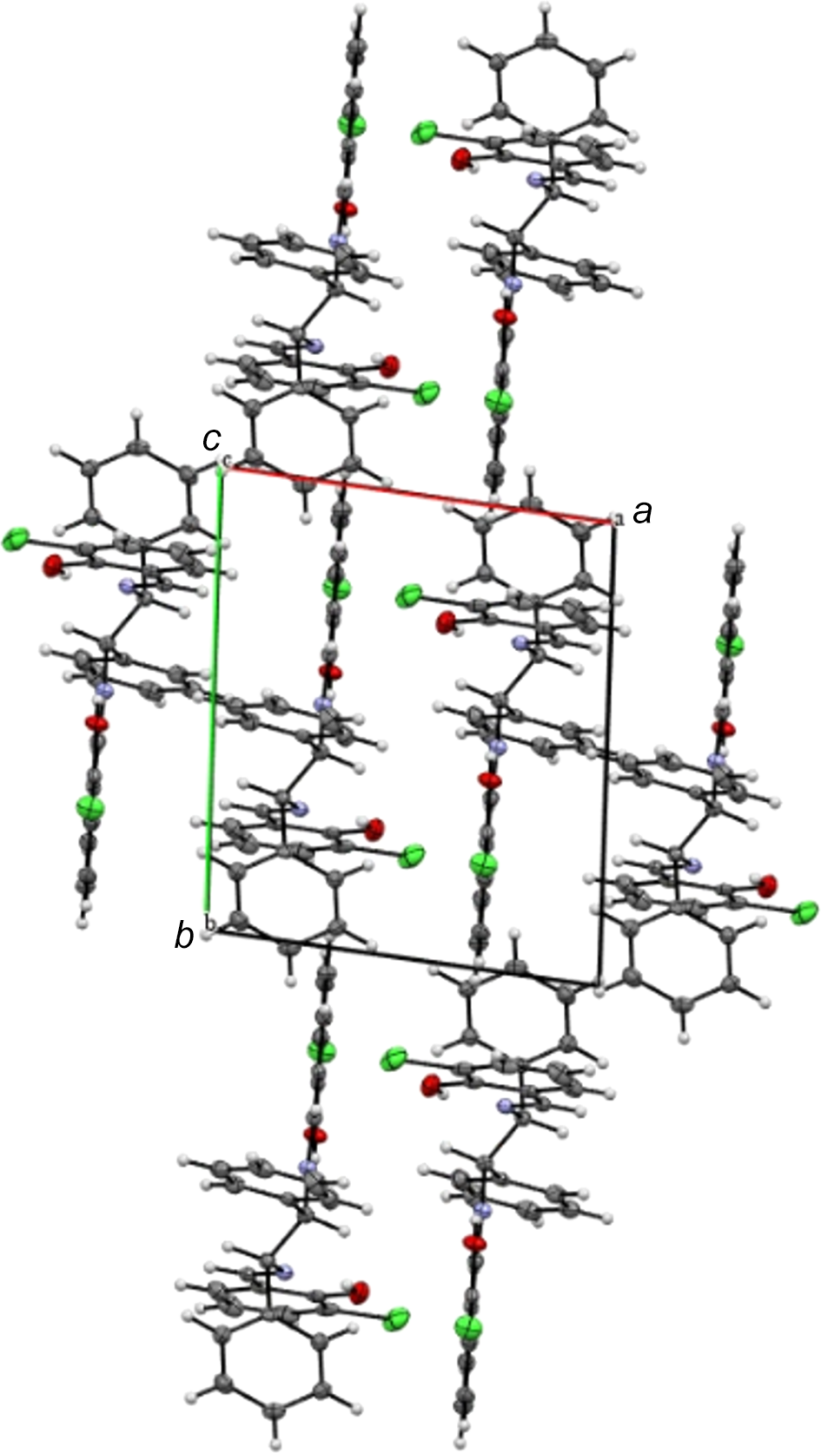
Packing of the title compound, viewed along the *b-*axis direction.

No inter­molecular hydrogen bonds are observed in the crystal, but a Cl⋯Cl halogen inter­action with length of 3.4122 (11) Å is found. There are two mol­ecules in the unit cell. In contrast, the unit cell of the monoclinic polymorph contains 12 mol­ecules, with an inter­molecular O⋯Cl hydrogen bond of 3.176 Å in length, weak O⋯H inter­actions (2.688 and 2.570 Å) and a C⋯Cl inter­action (3.225 Å; Shen *et al.*, 2017 ▸). An inter­esting feature of the crystal packing (Figs. 2 ▸–4 ▸ ▸) of the title compound is the presence of centrosymmetric Cl33⋯Cl34 [3.4122 (11) Å] and *n*⋯π^*^ (Echeverriá *et al.*, 2018 ▸) [O30⋯C22 = 3.029 (3) Å] inter­actions, which are shorter than sum of the van der Waals radii of the involved atoms (Bondi *et al.*, 1964 ▸). These inter­actions are supported by a short C4⋯C19 [3.361 (3) Å] contact, forming a one-dimensional extended chain of neighboring mol­ecules parallel to the *bc*-plane.

**Figure 3:**
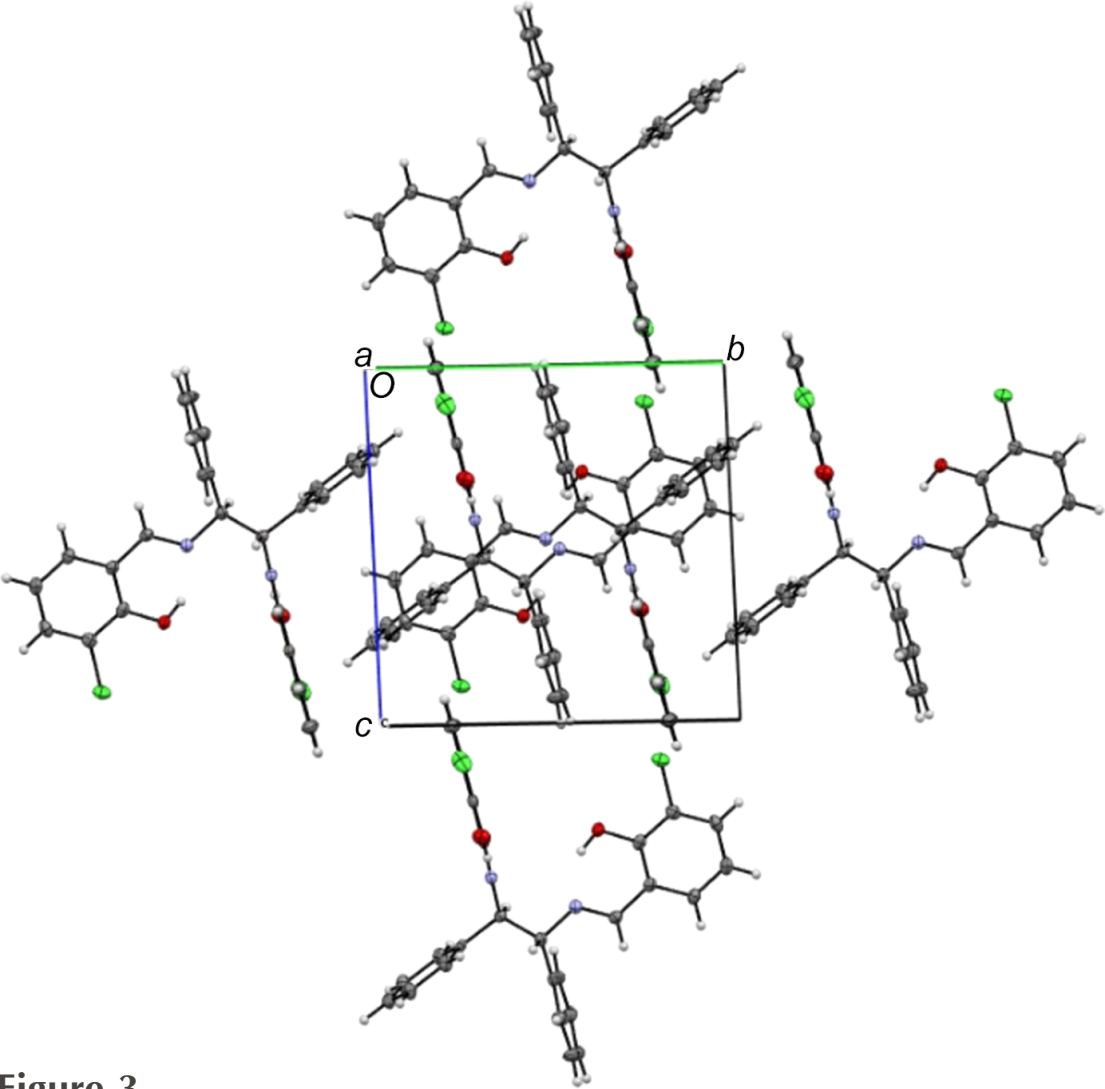
Packing of the title compound viewed along the *a-*axis direction.

**Figure 5:**
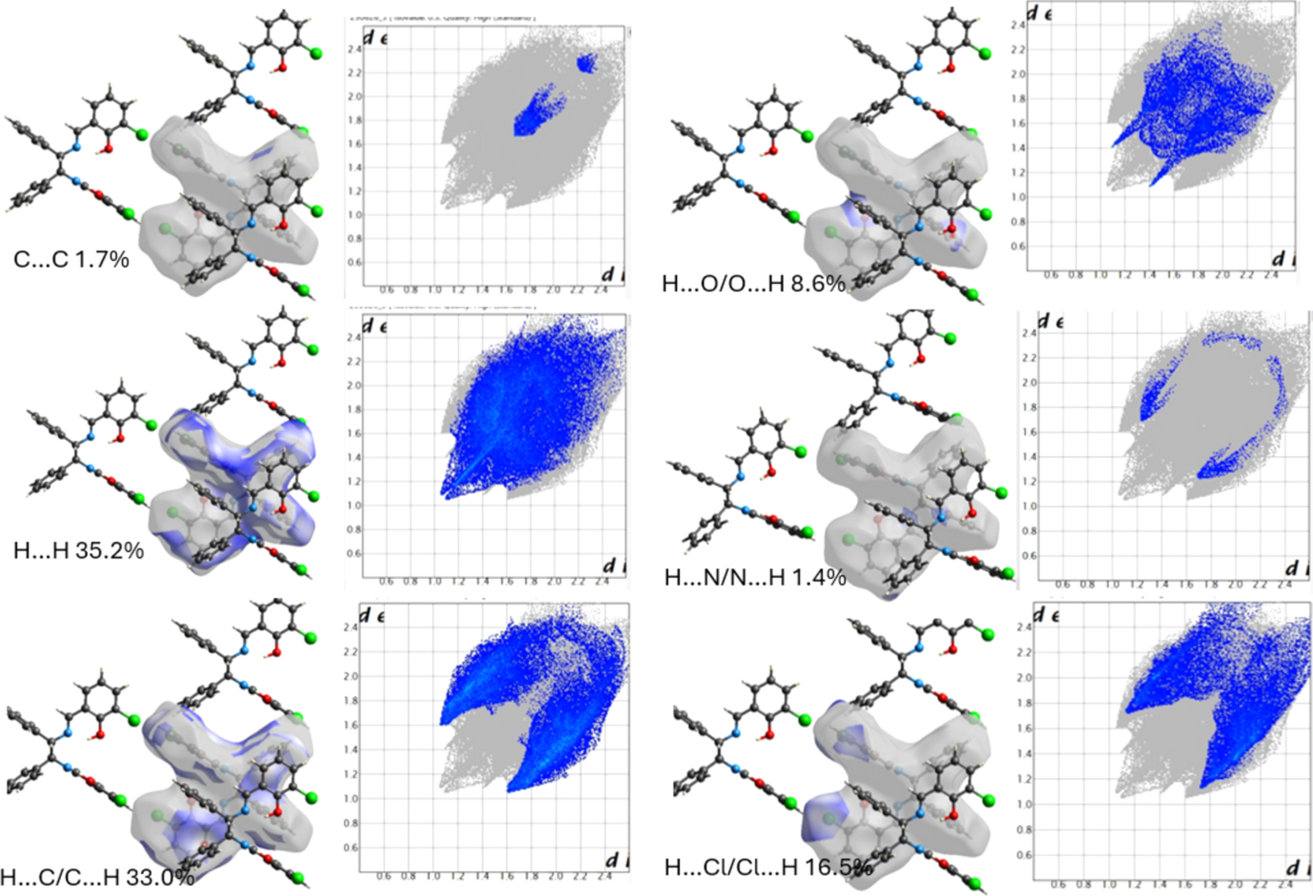
Hirshfeld surface mapped over *d*_norm_ and the two-dimensional fingerprint plots.

A Hirshfeld surface analysis (McKinnon *et al.*, 2004 ▸) was performed to further investigate the inter­molecular inter­actions and contacts using *Crystal Explorer 17.5* (McKinnon *et al.*, 2007 ▸; Turner *et al.*, 2017 ▸) (Fig. 5 ▸). It indicates that the most important contributions to the packing are from H⋯H (35.2%), C⋯H/H⋯C (18.4%) and Cl⋯H/H⋯Cl (10.3%) contacts. The inter­molecular C—H⋯C hydrogen bonds are indicated by bright-red spots on the Hirshfeld surfaces mapped over *d*_norm_ and by two sharp spikes of almost the same length in the region 1.6 Å < (*d*_e_ + *d*_i_) < 2.4 Å in the 2D finger plots (Fig. 3 ▸).

The contributions to the packing from H⋯H, C⋯C, C⋯H/H⋯C, Cl⋯H/H⋯Cl, N⋯H/H⋯N, and H⋯O/O⋯H contacts are 35.2, 1.7, 33.0, 16.5, 1.4 and 8.6%, respectively. The structure is characterized by a high proportion of H⋯H inter­actions, which are van der Waals inter­actions. The high C⋯H/H⋯C value is due to the presence of aromatic rings in the compound. The low C⋯C value is due to the lack of overlapping aromatic rings in the structure.

## 4. Database survey

A search in the Cambridge Structural Database (CSD, Version 5.41, update of January 2024; Groom *et al.* 2016 ▸) for similar structures gave 6,6′-{(1*E*,1*E*′)-[(1,2-di­phenyl­ethane-1,2-di­yl)bis­(aza­neylyl­idene)]bis­(methane­ylyl­idene)}bis­(2-fluoro­phenol), which is the monoclinic polymorph (refcode LECYUQ; Shen *et al.*, 2017 ▸). Besides this, there are several similar compounds, such as 6,6′-((1*E*,1*E*′)-{[(1*R*,2*R*)-1,2-di­phenyl­ethane-1,2-di­yl]bis­(aza­nylyl­idene)}bis­(methanylyl­idene))bis­(2-ethyl­phenol) (ref­code OWIJAI; Xu *et al.*, 2021 ▸) and *N*,*N*-sisalicyl­idene-(*R*,*S*)(*S*,*R*)-1,2-ethanedi­amine (refcode DSPEDN01; Ramazani *et al.*, 2006 ▸).

## 5. Synthesis and crystallization

3-Chloro­salicyl­aldehyde (0.039 g, 0.25 mmol) and *racemic*-(+/-)-1,2-di­phenyl­ethyl­enedi­amine (0.027 g, 0.125 mmol) were dissolved in 18 mL of methanol. The solution was stirred 50 s and irradiated with microwaves for 10 minutes at 353 K. The resulting clear yellow solution was evaporated. Recrystallized by slow evaporation of diethyl ether solution gave clear yellow rectangular-parallelepiped-shaped single crystals suitable for single-crystal X-ray diffraction analysis within a day. IR (ATR, cm ^−1^): 522 (*s*), 776 (*m*), 832 (*w*), 896 (*s*), 1093 (*m*, C—OH), 1413 (*w*), 1447 (*w*), 1624 (*m*, C=N)

## 6. Refinement

Crystal data, data collection and structure refinement details are summarized in Table 2 ▸. All C-bound H atoms were placed in geometrically calculated positions (C—H = 0.95–0.10 Å) and were refined using a riding model with *U*_iso_(H) = 1.2*U*_eq_(C) for *R*_2_CH and *R*_3_CH H atoms and 1.5*U*_eq_(C) for the methyl H atoms. The O-bound H atoms H29, H30 were located based on a difference-Fourier map and refined using constraints. In the process of removing the weak reciprocal lattice points due to twins, some reflections that should be present may have been inadvertently deleted.

## Supplementary Material

Crystal structure: contains datablock(s) global, I. DOI: 10.1107/S2056989025008850/ox2018sup1.cif

Structure factors: contains datablock(s) I. DOI: 10.1107/S2056989025008850/ox2018Isup2.hkl

Supporting information file. DOI: 10.1107/S2056989025008850/ox2018Isup3.cml

CCDC reference: 2494676

Additional supporting information:  crystallographic information; 3D view; checkCIF report

## Figures and Tables

**Figure 4:**
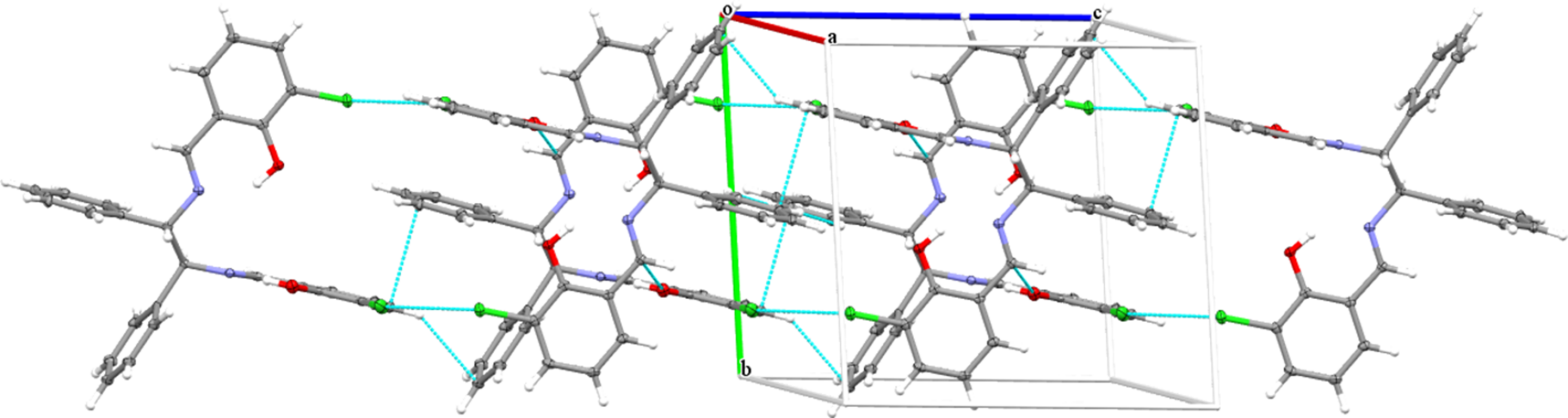
The packing of the title compound, showing the chain formed by Cl⋯Cl and O⋯C contacts.

**Table 1:** Hydrogen-bond geometry (Å, °)

*D*—H⋯*A*	*D*—H	H⋯*A*	*D*⋯*A*	*D*—H⋯*A*
O29—H29⋯N31	0.84	1.82	2.564 (3)	147
O30—H30⋯N32	0.84	1.85	2.597 (3)	147

**Table 2:** Experimental details

Crystal data	
Chemical formula	C_28_H_22_Cl_2_N_2_O_2_
*M*_r_	489.37
Crystal system, space group	Triclinic, *P* 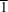
Temperature (K)	90
*a*, *b*, *c* (Å)	9.8543 (15), 11.1140 (17), 11.4288 (18)
α, β, γ (°)	86.563 (5), 74.417 (5), 82.951 (5)
*V* (Å^3^)	1196.1 (3)
*Z*	2
Radiation type	Mo *K*α
μ (mm^−1^)	0.30
Crystal size (mm)	0.80 × 0.30 × 0.30

Data collection	
Diffractometer	Bruker D8 QUEST
Absorption correction	Multi-scan (*SADABS*; Krause *et al.*, 2015 ▸)
*T*_min_, *T*_max_	0.70, 0.92
No. of measured, independent and observed [*I* > 2σ(*I*)] reflections	7500, 2909, 2808
*R*_int_	0.057
θ_max_ (°)	22.4
(sin θ/λ)_max_ (Å^−1^)	0.536

Refinement	
*R*[*F*^2^ > 2σ(*F*^2^)], *wR*(*F*^2^), *S*	0.078, 0.178, 1.14
No. of reflections	2909
No. of parameters	309
H-atom treatment	H atoms treated by a mixture of independent and constrained refinement
Δρ_max_, Δρ_min_ (e Å^−3^)	0.78, −0.56

Computer programs: *APEX4* and *SAINT* (Bruker, 2019 ▸), *SHELXT2018/2* (Sheldrick, 2015*a* ▸), *SHELXL2019/1* (Sheldrick, 2015*b* ▸) and *shelXleShelXle* (Hübschle *et al.*, 2011 ▸).

## supplementary crystallographic information

### 6,6'-{(1E,1E')-[(1,2-Diphenylethane-1,2-diyl)bis(azaneylylidene)]bis(methaneylylidene)}bis(2-chlorophenol) Crystal data

**Table:**

C_28_H_22_Cl_2_N_2_O_2_	*Z* = 2
*M**_r_* = 489.37	*F*(000) = 508
Triclinic, *P*1	*D*_x_ = 1.359 Mg m^−^^3^
*a* = 9.8543 (15) Å	Mo *K*α radiation, λ = 0.71073 Å
*b* = 11.1140 (17) Å	Cell parameters from 7076 reflections
*c* = 11.4288 (18) Å	θ = 2.6–22.4°
α = 86.563 (5)°	µ = 0.30 mm^−^^1^
β = 74.417 (5)°	*T* = 90 K
γ = 82.951 (5)°	Plate, yellow
*V* = 1196.1 (3) Å^3^	0.80 × 0.30 × 0.30 mm

### 6,6'-{(1E,1E')-[(1,2-Diphenylethane-1,2-diyl)bis(azaneylylidene)]bis(methaneylylidene)}bis(2-chlorophenol) Data collection

**Table:**

Bruker D8 QUEST diffractometer	2808 reflections with *I* > 2σ(*I*)
Detector resolution: 7.3910 pixels mm^-1^	*R*_int_ = 0.057
φ and ω scans	θ_max_ = 22.4°, θ_min_ = 1.9°
Absorption correction: multi-scan (SADABS; Krause *et al.*, 2015)	*h* = −10→10
*T*_min_ = 0.70, *T*_max_ = 0.92	*k* = −11→11
7500 measured reflections	*l* = −12→12
2909 independent reflections	

### 6,6'-{(1E,1E')-[(1,2-Diphenylethane-1,2-diyl)bis(azaneylylidene)]bis(methaneylylidene)}bis(2-chlorophenol) Refinement

**Table:**

Refinement on *F*^2^	0 restraints
Least-squares matrix: full	Hydrogen site location: inferred from neighbouring sites
*R*[*F*^2^ > 2σ(*F*^2^)] = 0.078	H atoms treated by a mixture of independent and constrained refinement
*wR*(*F*^2^) = 0.178	*w* = 1/[σ^2^(*F*_o_^2^) + (0.1181*P*)^2^ + 0.4041*P*] where *P* = (*F*_o_^2^ + 2*F*_c_^2^)/3
*S* = 1.14	(Δ/σ)_max_ < 0.001
2909 reflections	Δρ_max_ = 0.78 e Å^−^^3^
309 parameters	Δρ_min_ = −0.56 e Å^−^^3^

### 6,6'-{(1E,1E')-[(1,2-Diphenylethane-1,2-diyl)bis(azaneylylidene)]bis(methaneylylidene)}bis(2-chlorophenol) Special details

**Table:**

Geometry. All esds (except the esd in the dihedral angle between two l.s. planes) are estimated using the full covariance matrix. The cell esds are taken into account individually in the estimation of esds in distances, angles and torsion angles; correlations between esds in cell parameters are only used when they are defined by crystal symmetry. An approximate (isotropic) treatment of cell esds is used for estimating esds involving l.s. planes.

### 6,6'-{(1E,1E')-[(1,2-Diphenylethane-1,2-diyl)bis(azaneylylidene)]bis(methaneylylidene)}bis(2-chlorophenol) Fractional atomic coordinates and isotropic or equivalent isotropic displacement parameters (Å^2^)

**Table:**

	*x*	*y*	*z*	*U*_iso_*/*U*_eq_	
Cl33	0.69894 (7)	0.77330 (6)	0.10865 (5)	0.0321 (3)	
Cl34	0.48781 (7)	0.21930 (6)	0.10402 (6)	0.0386 (3)	
O29	0.70703 (17)	0.59076 (14)	0.30123 (15)	0.0236 (5)	
H29	0.707911	0.541261	0.359594	0.068 (12)*	
O30	0.58282 (17)	0.26535 (16)	0.31484 (15)	0.0267 (5)	
H30	0.614604	0.279211	0.373340	0.066 (12)*	
N31	0.72307 (19)	0.51873 (17)	0.51494 (17)	0.0183 (5)	
N32	0.77316 (19)	0.28700 (16)	0.42902 (17)	0.0183 (5)	
C1	0.9058 (3)	0.4613 (2)	0.7130 (2)	0.0220 (6)	
H1	0.977177	0.452213	0.638356	0.026000*	
C12	0.7867 (3)	−0.0137 (2)	0.7700 (2)	0.0238 (6)	
H12	0.779742	−0.084463	0.821244	0.029000*	
C13	0.6670 (3)	0.0463 (2)	0.7423 (2)	0.0234 (6)	
H13	0.577598	0.016854	0.775207	0.028000*	
C14	0.6770 (2)	0.1492 (2)	0.6668 (2)	0.0214 (6)	
H14	0.594582	0.189105	0.647407	0.026000*	
C15	0.8672 (2)	0.2710 (2)	0.3286 (2)	0.0179 (6)	
H15	0.963560	0.274867	0.326773	0.021000*	
C16	0.8330 (3)	0.24711 (19)	0.2164 (2)	0.0195 (6)	
C17	0.9410 (3)	0.2246 (2)	0.1100 (2)	0.0264 (6)	
H17	1.036817	0.227321	0.110552	0.032000*	
C18	0.9104 (3)	0.1985 (2)	0.0043 (2)	0.0325 (7)	
H18	0.984757	0.182025	−0.067299	0.039000*	
C19	0.7710 (3)	0.1964 (2)	0.0027 (2)	0.0307 (7)	
H19	0.749397	0.178877	−0.070156	0.037000*	
C20	0.6632 (3)	0.2197 (2)	0.1067 (2)	0.0253 (6)	
C21	0.6908 (3)	0.24488 (19)	0.2153 (2)	0.0207 (6)	
C22	0.7105 (2)	0.6290 (2)	0.5464 (2)	0.0180 (6)	
H22	0.705884	0.646557	0.627801	0.022000*	
C23	0.7031 (2)	0.7283 (2)	0.4572 (2)	0.0174 (6)	
C24	0.6948 (2)	0.8487 (2)	0.4918 (2)	0.0215 (6)	
H24	0.691537	0.864924	0.573228	0.026000*	
C25	0.6914 (2)	0.9436 (2)	0.4090 (2)	0.0236 (6)	
H25	0.685739	1.024745	0.433281	0.028000*	
C26	0.6962 (2)	0.9203 (2)	0.2899 (2)	0.0236 (6)	
H26	0.695707	0.985296	0.232084	0.028000*	
C27	0.7018 (2)	0.8019 (2)	0.2556 (2)	0.0216 (6)	
C28	0.7046 (2)	0.7043 (2)	0.3380 (2)	0.0186 (6)	
C2	0.9419 (3)	0.4896 (2)	0.8163 (2)	0.0248 (6)	
H2	1.037668	0.498182	0.812667	0.030000*	
C3	0.8383 (3)	0.5054 (2)	0.9247 (2)	0.0274 (6)	
H3	0.862207	0.526466	0.995457	0.033000*	
C4	0.6999 (3)	0.4904 (2)	0.9294 (2)	0.0304 (7)	
H4	0.628617	0.500581	1.003883	0.036000*	
C5	0.6643 (3)	0.4606 (2)	0.8265 (2)	0.0261 (6)	
H5	0.568705	0.450108	0.831112	0.031000*	
C6	0.7664 (2)	0.4459 (2)	0.7168 (2)	0.0185 (6)	
C7	0.7240 (2)	0.4172 (2)	0.6047 (2)	0.0181 (6)	
H7	0.625043	0.394426	0.632597	0.022000*	
C8	0.8179 (2)	0.3074 (2)	0.5376 (2)	0.0167 (6)	
H8	0.918827	0.325516	0.512654	0.020000*	
C9	0.8068 (2)	0.1941 (2)	0.6193 (2)	0.0171 (6)	
C10	0.9253 (3)	0.1342 (2)	0.6481 (2)	0.0236 (6)	
H10	1.014514	0.164461	0.616680	0.028000*	
C11	0.9160 (3)	0.0300 (2)	0.7228 (2)	0.0270 (6)	
H11	0.998589	−0.010831	0.741072	0.032000*	

### 6,6'-{(1E,1E')-[(1,2-Diphenylethane-1,2-diyl)bis(azaneylylidene)]bis(methaneylylidene)}bis(2-chlorophenol) Atomic displacement parameters (Å^2^)

**Table:**

	*U*^11^	*U*^22^	*U*^33^	*U*^12^	*U*^13^	*U*^23^
Cl33	0.0450 (5)	0.0330 (5)	0.0197 (5)	−0.0019 (3)	−0.0125 (3)	0.0016 (3)
Cl34	0.0431 (5)	0.0379 (5)	0.0479 (6)	−0.0175 (4)	−0.0315 (4)	0.0108 (4)
O29	0.0337 (10)	0.0160 (9)	0.0221 (10)	−0.0004 (7)	−0.0094 (8)	−0.0029 (8)
O30	0.0221 (10)	0.0335 (11)	0.0255 (11)	−0.0067 (8)	−0.0072 (8)	0.0025 (8)
N31	0.0189 (10)	0.0160 (11)	0.0199 (11)	−0.0015 (8)	−0.0048 (9)	−0.0009 (8)
N32	0.0209 (11)	0.0132 (10)	0.0207 (11)	−0.0014 (8)	−0.0053 (9)	0.0002 (8)
C1	0.0250 (13)	0.0214 (13)	0.0189 (13)	−0.0035 (10)	−0.0039 (11)	−0.0020 (10)
C12	0.0317 (14)	0.0166 (13)	0.0244 (14)	−0.0037 (11)	−0.0098 (11)	0.0019 (10)
C13	0.0229 (13)	0.0196 (13)	0.0288 (14)	−0.0063 (10)	−0.0076 (11)	0.0008 (10)
C14	0.0198 (13)	0.0190 (13)	0.0270 (14)	−0.0017 (10)	−0.0090 (11)	−0.0009 (10)
C15	0.0178 (12)	0.0128 (12)	0.0218 (14)	−0.0009 (9)	−0.0035 (11)	−0.0004 (9)
C16	0.0263 (13)	0.0103 (12)	0.0221 (14)	−0.0002 (10)	−0.0075 (11)	0.0006 (9)
C17	0.0291 (14)	0.0243 (14)	0.0241 (15)	0.0036 (11)	−0.0067 (12)	−0.0030 (11)
C18	0.0443 (17)	0.0313 (15)	0.0205 (14)	0.0075 (12)	−0.0101 (12)	−0.0057 (11)
C19	0.0528 (18)	0.0196 (14)	0.0248 (15)	0.0010 (12)	−0.0204 (13)	−0.0040 (11)
C20	0.0388 (16)	0.0120 (12)	0.0311 (15)	−0.0058 (11)	−0.0191 (13)	0.0036 (10)
C21	0.0275 (14)	0.0094 (12)	0.0255 (14)	−0.0021 (10)	−0.0084 (11)	0.0029 (10)
C22	0.0150 (12)	0.0199 (13)	0.0184 (13)	−0.0014 (9)	−0.0031 (10)	−0.0027 (10)
C23	0.0126 (11)	0.0177 (12)	0.0205 (13)	0.0006 (9)	−0.0026 (10)	−0.0020 (10)
C24	0.0191 (13)	0.0194 (13)	0.0260 (14)	−0.0001 (10)	−0.0061 (10)	−0.0034 (10)
C25	0.0231 (13)	0.0164 (13)	0.0317 (15)	−0.0025 (10)	−0.0076 (11)	−0.0015 (10)
C26	0.0201 (12)	0.0187 (13)	0.0311 (15)	−0.0008 (10)	−0.0073 (11)	0.0058 (10)
C27	0.0183 (12)	0.0242 (14)	0.0220 (14)	−0.0007 (10)	−0.0059 (10)	0.0012 (10)
C28	0.0117 (12)	0.0190 (13)	0.0244 (14)	0.0003 (9)	−0.0038 (10)	−0.0044 (10)
C2	0.0293 (14)	0.0189 (13)	0.0280 (15)	−0.0028 (11)	−0.0106 (12)	−0.0011 (10)
C3	0.0413 (17)	0.0211 (13)	0.0215 (14)	0.0039 (11)	−0.0141 (12)	−0.0028 (10)
C4	0.0343 (16)	0.0372 (15)	0.0156 (13)	0.0053 (12)	−0.0030 (11)	−0.0049 (11)
C5	0.0229 (13)	0.0287 (14)	0.0232 (14)	0.0006 (11)	−0.0019 (11)	−0.0006 (11)
C6	0.0239 (13)	0.0108 (12)	0.0191 (13)	0.0007 (9)	−0.0040 (10)	0.0009 (9)
C7	0.0176 (12)	0.0160 (12)	0.0203 (13)	−0.0027 (9)	−0.0045 (10)	0.0004 (10)
C8	0.0161 (12)	0.0172 (12)	0.0171 (13)	−0.0021 (9)	−0.0046 (10)	−0.0015 (9)
C9	0.0203 (12)	0.0144 (12)	0.0165 (12)	−0.0005 (9)	−0.0043 (10)	−0.0063 (9)
C10	0.0193 (13)	0.0210 (14)	0.0302 (14)	−0.0032 (10)	−0.0050 (11)	−0.0019 (11)
C11	0.0248 (14)	0.0226 (14)	0.0351 (16)	0.0024 (11)	−0.0132 (12)	0.0013 (11)

### 6,6'-{(1E,1E')-[(1,2-Diphenylethane-1,2-diyl)bis(azaneylylidene)]bis(methaneylylidene)}bis(2-chlorophenol) Geometric parameters (Å, º)

**Table:**

Cl33—C27	1.736 (2)	C19—C20	1.378 (4)
Cl34—C20	1.738 (3)	C20—C21	1.390 (4)
O29—C28	1.349 (3)	C22—C23	1.465 (3)
O30—C21	1.341 (3)	C23—C28	1.399 (3)
N31—C22	1.280 (3)	C23—C24	1.405 (3)
N31—C7	1.479 (3)	C24—C25	1.378 (4)
N32—C15	1.273 (3)	C25—C26	1.388 (4)
N32—C8	1.463 (3)	C26—C27	1.386 (4)
C1—C2	1.385 (4)	C27—C28	1.396 (4)
C1—C6	1.394 (4)	C2—C3	1.382 (4)
C12—C11	1.379 (4)	C3—C4	1.381 (4)
C12—C13	1.385 (4)	C4—C5	1.383 (4)
C13—C14	1.388 (4)	C5—C6	1.385 (4)
C14—C9	1.390 (3)	C6—C7	1.513 (3)
C15—C16	1.457 (3)	C7—C8	1.545 (3)
C16—C17	1.397 (4)	C8—C9	1.519 (3)
C16—C21	1.409 (4)	C9—C10	1.381 (3)
C17—C18	1.376 (4)	C10—C11	1.394 (4)
C18—C19	1.382 (4)		
			
C22—N31—C7	121.14 (19)	C27—C26—C25	119.8 (2)
C15—N32—C8	118.73 (19)	C26—C27—C28	121.4 (2)
C2—C1—C6	121.0 (2)	C26—C27—Cl33	119.82 (19)
C11—C12—C13	119.5 (2)	C28—C27—Cl33	118.69 (18)
C12—C13—C14	120.3 (2)	O29—C28—C27	119.4 (2)
C13—C14—C9	120.5 (2)	O29—C28—C23	122.2 (2)
N32—C15—C16	122.6 (2)	C27—C28—C23	118.4 (2)
C17—C16—C21	119.7 (2)	C3—C2—C1	119.9 (2)
C17—C16—C15	120.3 (2)	C4—C3—C2	119.5 (2)
C21—C16—C15	120.1 (2)	C3—C4—C5	120.6 (2)
C18—C17—C16	120.8 (2)	C4—C5—C6	120.7 (2)
C17—C18—C19	119.7 (2)	C5—C6—C1	118.3 (2)
C20—C19—C18	120.1 (2)	C5—C6—C7	119.7 (2)
C19—C20—C21	121.6 (2)	C1—C6—C7	122.0 (2)
C19—C20—Cl34	120.1 (2)	N31—C7—C6	115.14 (18)
C21—C20—Cl34	118.4 (2)	N31—C7—C8	107.84 (18)
O30—C21—C20	119.6 (2)	C6—C7—C8	112.28 (19)
O30—C21—C16	122.2 (2)	N32—C8—C9	109.64 (17)
C20—C21—C16	118.1 (2)	N32—C8—C7	109.18 (18)
N31—C22—C23	120.2 (2)	C9—C8—C7	110.89 (18)
C28—C23—C24	119.8 (2)	C10—C9—C14	118.8 (2)
C28—C23—C22	120.7 (2)	C10—C9—C8	120.6 (2)
C24—C23—C22	119.6 (2)	C14—C9—C8	120.6 (2)
C25—C24—C23	120.8 (2)	C9—C10—C11	120.9 (2)
C24—C25—C26	119.8 (2)	C12—C11—C10	120.0 (2)

### 6,6'-{(1E,1E')-[(1,2-Diphenylethane-1,2-diyl)bis(azaneylylidene)]bis(methaneylylidene)}bis(2-chlorophenol) Hydrogen-bond geometry (Å, º)

**Table:**

*D*—H···*A*	*D*—H	H···*A*	*D*···*A*	*D*—H···*A*
O29—H29···N31	0.84	1.82	2.564 (3)	147
O30—H30···N32	0.84	1.85	2.597 (3)	147
